# Characterization and Analysis of Porosities in High Pressure Die Cast Aluminum by Using Metallography, X-Ray Radiography, and Micro-Computed Tomography

**DOI:** 10.3390/ma13143068

**Published:** 2020-07-09

**Authors:** Ahmad Nourian-Avval, Ali Fatemi

**Affiliations:** Department of Mechanical Engineering, University of Memphis, Memphis, TN 38152, USA; nrnavval@memphis.edu

**Keywords:** high pressure die casting, defect characterization, extreme value statistics, aluminum castings

## Abstract

Mechanical performance of cast aluminum alloys is strongly affected by the defects formed during solidification. For example, fractography studies of the fatigue specimens have shown that fatigue failure in aluminum castings containing defects is almost always initiated from defects, among which pores are most detrimental. However, elimination of these pores is neither always economically nor technically possible. This work characterizes defects in high pressure die cast aluminum alloy as an illustrative material, but the methods used can be applicable to other types of castings and defects. The defects were evaluated using metallography as well as micro-computed tomography techniques. The variability of defects between the specimens of two sizes as well as different porosity levels are studied statistically. The distributions of defects based on location within the specimens are also analyzed. Moreover, the maximum defect size within the specimens are estimated using extreme value statistics, which can be used as an input to fatigue life prediction models. Extreme value statistics is applied on both 2D and 3D defect data. The accuracy of each approach is verified by comparing the estimated maximum defect size within the specimens with the maximum observed defects on fracture surfaces of fatigue specimens.

## 1. Introduction

Cast aluminum alloys are increasingly used in producing a variety of components in different industries including aerospace, automotive, electrical, and railroad. The wide range of application of these alloys is due to their excellent castability, corrosion resistance, and especially high strength to weight ratio which increases performance and fuel efficiency [1,2]. One of the most productive casting methods in producing a wide variety of aluminum components with high dimensional accuracy and complex geometries is high pressure die casting (HPDC). However, the melt in HPDC is more turbulent during mold filling, which may result in substantial porosity due to entrapped air [2]. 

In addition to entrapped air, porosities can also be attributed to dissolving hydrogen released from the reaction of water vapor and melt at high temperature and being expelled during solidification. Porosities may also result from shrinkage due to natural volume contraction of the melt [2,3]. Oxide films represent another type of defects which is common in cast components. These oxide films may be formed during mold filling by surface turbulence or come from the crucible [4,5]. All these defects can result in significantly reducing mechanical properties such as strength and ductility in general, and fatigue performance in particular. Casting defects not only reduce the fatigue strength, but also cause significant scatter in fatigue life [6,7,8,9]. Such defects give rise to stress concentrations that promote localized damage by inducing crack formation and growth [10,11,12].

Boileau et al. [13,14] found that fatigue failure of cast aluminum alloys containing porosity is independent of microstructure, and is almost always initiated from the pores. Mayer et al. [15] found that 98.5% of fatigue failures of cast magnesium and cast aluminum alloys all produced by HPDC initiated from porosities. Wang et al. [16] investigated the fatigue behavior of A356-T6 alloy and demonstrated that pores were more detrimental to fatigue life than oxides. Studies on A356 alloy show that the elimination of porosity can increase the average fatigue life by several orders of magnitude [4,14,17]. In the absence of porosity, fatigue crack initiation occurred from microstructural features such as intermetallic phases and slip bands [18,19,20]. However, in presence of pores, the crack initiation life is negligible and most of the fatigue life is spent on crack growth [14,19,21,22].

In spite of great effort dedicated to reduce porosity within cast components, complete elimination of defects is neither technically nor economically possible [23]. This necessitates defect characterization. For defect characterization, size, density, location, shape, and orientation of defects can be considered. Defect size perhaps is the most significant parameter affecting the fatigue performance since it has direct influence on local stress and strain concentrations during cyclic loading. Finite element analysis (FEA) [24,25] as well as experimental evidence suggest that the largest defects at or near the surface of cast components are most detrimental to fatigue performance [26,27,28]. Seniw et al. [27] showed that for pores of comparable size, the fatigue life increased with their distance from the free surface, and large pores far from the free surface had less detrimental effect on fatigue life compared to smaller pores located near the free surface. 

Regarding the shape and orientation of defects, those with complex shapes containing arcs with sharp root radii are the preferred sites for fatigue crack initiation as opposed to those with a more spherical shape [29]. However, FEA analysis by Gao et al. [24] showed that the maximum stress concentration around a pore with a complex shape was only 3% higher than that with a simpler shape but equivalent projected area and local curvature radii. Buffiere et al. [30] also reported only a 10% difference between the stress intensity factor of a pore with a complex shape and that of elliptical one with the same projected defect size. Furthermore, Li et al. [31] suggested that the stress and strain concentrations are significantly affected by the projected area on the transverse plane to the loading direction, rather than the local radius of curvature of defects. Therefore, shape and orientation effects may be insignificant if there is no change in the effective size of the pores and their local curvature radii.

Characterization of the defects may be performed non-destructively by X-ray micro-computed tomography (μCT) [32,33,34] or destructively using metallography examination [35,36]. X-ray μCT has the capability of evaluating the pores in a three-dimensional scale, but it is costly and not always accessible. On the other hand, metallography examination provides two-dimensional information of defects on polished cross sections, while the measurements obtained from random sections highly depend on where the random section is made. Moreover, the definition of the equivalent size of pores is not straightforward as it is affected by the pore morphology which depends on the type of porosity. Gas pores are typically spherical and, therefore, appear as rounded shapes on a metallographic cross-section, while shrinkage porosities are interdendritic cavities with branches that can appear with a different complex forms on a metallography section [32].

One objective of this paper was to compare the capabilities of metallography as well as X-ray radiography and micro-computed tomography techniques in defect characterization and analysis of their variability in high pressure die cast aluminum, as an illustrative material containing defects. On the other hand, in evaluation of defects in castings it is often assumed that defects are distributed uniformly in the entire cross section, while this may not be true in all cases. Therefore, in this study pore distributions in specimens with different sizes and porosity levels are compared, and the variability of defects based on location within the specimens is analyzed. Furthermore, the maximum defect size within the specimens is estimated by extreme value statistics using the evaluated defect data from both metallography and µCT scan. The accuracy of each approach is verified by comparing the estimated maximum defects with the maximum defect size observed on fracture surfaces. The estimated maximum defect size within the specimens will be the input for fatigue life prediction models to predict the fatigue life under different loading condition, which are discussed in another work by the authors [37].

## 2. Extreme Value Statistics for Defect Analysis

For defect evaluation and estimation of the maximum defect size possible within the specimens or components, extreme value statistics (EVS) can be used. Using metallography evaluation, Murakami et al. [38] proposed an experimental procedure based on block maxima (BM) approach to estimate the largest defect expected in a volume of material. Based on this procedure, a statistically significant number of separate regions (control areas), each of the same area, *S_0_*, within metallography cross sections are analyzed and the largest defect size in each control area is determined. Having obtained the cumulative probability distribution of the defects, the extreme value (EV) distribution function is fitted to determine the location and scale parameters, *µ* and *σ*. The EV distribution function is given by:

(1) $$F\left(x\right)=exp\left[-exp\left(-\frac{x-\mu }{\sigma }\right)\right]$$

Then, the characteristic largest defect, which is the maximum defect expected to be exceeded once in the desired volume, is the defect size corresponding to the return period, given as:

(2) $$T=\frac{V}{S_{0}h}$$

in which V is the volume of the specimen or component, $S_{0}$ is the control area, and h is the average value of the maximum defects from different inspected areas. It is noteworthy that Equation (1) can be rearranged to a linear form, as follows:

(3) $$-\ln\left(-\ln\left(F\left(x\right)\right)\right)=\frac{x}{\sigma }-\frac{\mu }{\sigma }$$

Therefore, when the cumulative probability distribution of defects is plotted as −ln(−ln(F(x))) vs. the equivalent defect size, the data points should be aligned on a straight line. However, it has been shown that in the case of cast aluminum alloys, the defect data points do not always align on a straight line [6,11,23,39]. 

It has been shown that generalized extreme value (GEV) distribution function gives rise to a better fit to pore size distribution in cast aluminum alloys [40]. The GEV distribution function is given by:

(4) $$F\left(x\right)=exp\left\{-{\left[1+\xi \left(\frac{x-\mu }{\sigma }\right)\right]}^{-\frac{1}{\xi }}\right\}$$

where *µ*, σ, and ξ are location, scale, and shape parameters, respectively. In fact, EV distribution function is a special case of GEV function when the shape parameter approaches to zero. However, the advantage of using GEV is that the data themselves will determine the most appropriate value for shape parameter.

On the other hand, for estimating the maximum defect size based on defect size data obtained through X-ray µCT, the peak-over-threshold (POT) approach can be used. In this approach, all the defects within the scanned volume above a given threshold are considered, therefore, no important information is lost. The generalized Pareto distribution (GPD) function is then fitted to the cumulative probability distribution of defects, given by:

(5) $$F\left(x\right)=1-{\left(1+\gamma \frac{x-u}{\sigma }\right)}^{-1/\gamma }$$

where γ, σ, and u are shape, scale, and location parameters, respectively. The maximum defect size is, then, the one attributed to the return period, T, which for the prospective material volume, V, containing a constant defect density, $\rho_{d}$, is given by:

(6) $$T=\rho_{d}V.$$

## 3. Materials and Methods 

**Figure 1:**
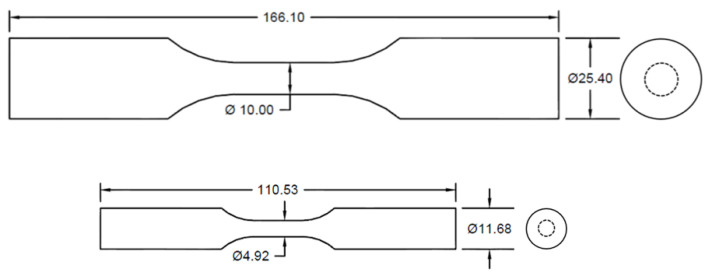
Illustration of the two specimen geometries used.

The material studied in this work was A356 aluminum alloy. Two specimen configurations with the nominal gauge section diameters of 5 mm and 10 mm were produced by the high pressure die casting method. The geometries of the specimens are illustrated in Figure 1. Initial evaluation of defects was carried out using X-ray radiography, through which the specimens were classified into four porosity levels with regards to the maximum detected defects size. Porosity level one contains the smallest porosities, while porosity level four has the largest detected porosities. The ranges of the maximum defects in different porosity levels are shown in Table 1. Defect evaluation and statistical analysis based on 2D and 3D defect data are compared by conducting metallography and X-ray micro-computed tomography scan, respectively. The volume fraction of defects in small and large specimens measured from µCT scan results at different porosity levels is also compared in Table 1. 

### 3.1. Defect Evaluation Using Metallography Examination

**Figure 2:**
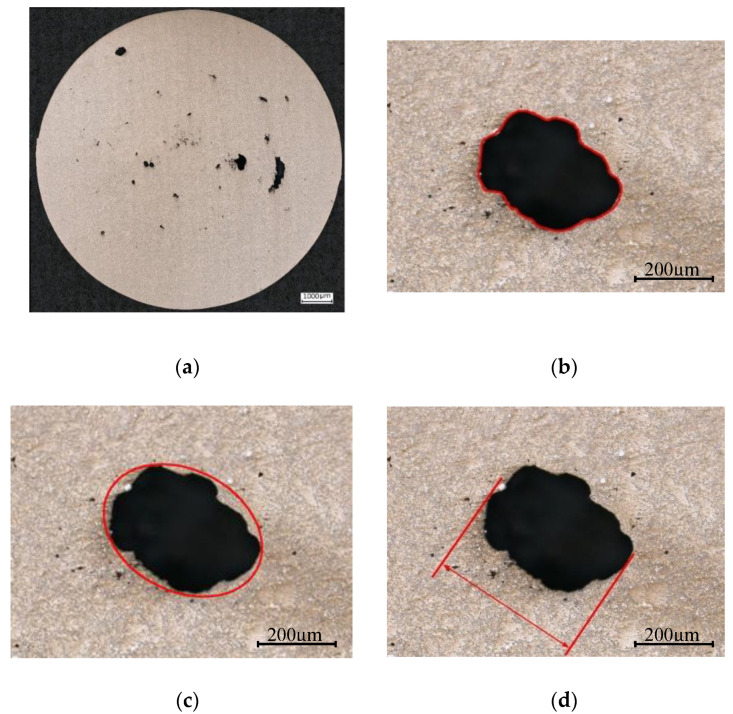
(**a**) an example of optical micrograph in a large specimen porosity level 3, three different defect parameters to determine the size of the defects, (**b**) actual area of the defect ($\sqrt{A_{actual}}$), (**c**) the area of fitted ellipse ($\sqrt{A_{Ellipse}}$), and (**d**) max Feret diameter ($Feret_{max}$).

Metallography is an accessible and cost-effective approach, which is commonly used to study the porosity of castings and compare the casting processes. However, evaluating the defects using metallography examination highly depends on where the random section is made. Therefore, increasing the number of cross sections will increase the reliability of the measurements and the measured defect population will better reflect the defect population within the specimens or components. For this reason, eight equally-spaced surfaces throughout the gauge length of the specimens were prepared for each specimen size and at each porosity level. Then, each cross section was polished and examined by a VHX–6000 series digital microscope capable of automatically stitching up to 200 pictures taken from the surface in magnification of 500x. Therefore, high resolution micrographs of the whole cross sections were prepared. This procedure was followed for three small specimens at each porosity level and two large specimens at porosity levels one and three. An example micrograph prepared from large specimen porosity level three is shown in Figure 2a. 

Having examined the polished surfaces using optical microscope, the defects information was extracted from the metallography micrographs using open source image analyzer software, ImageJ. Three different parameters were used to characterize the defects, the square root of actual area of defects, the square root of the ellipse fitted to each defect, and the maximum Feret diameter. These three defect parameters are demonstrated in Figure 2b–d. The comparison of defect distribution using these three defect parameters is discussed in Section 4.

### 3.2. Defect Evaluation Using X-ray Micro-Computed Tomography

X-ray micro-computed tomography is a powerful imaging technique, which is increasingly used in defect evaluation of castings. In this method, the specimen is exposed to a high density X-ray beam from different directions and the internal structure of the specimen is reconstructed through synthesizing the stack of individual projections in different directions. Therefore, the defects can be evaluated in a large material volume in 3D scale without need for specimen preparation. 

**Figure 3:**
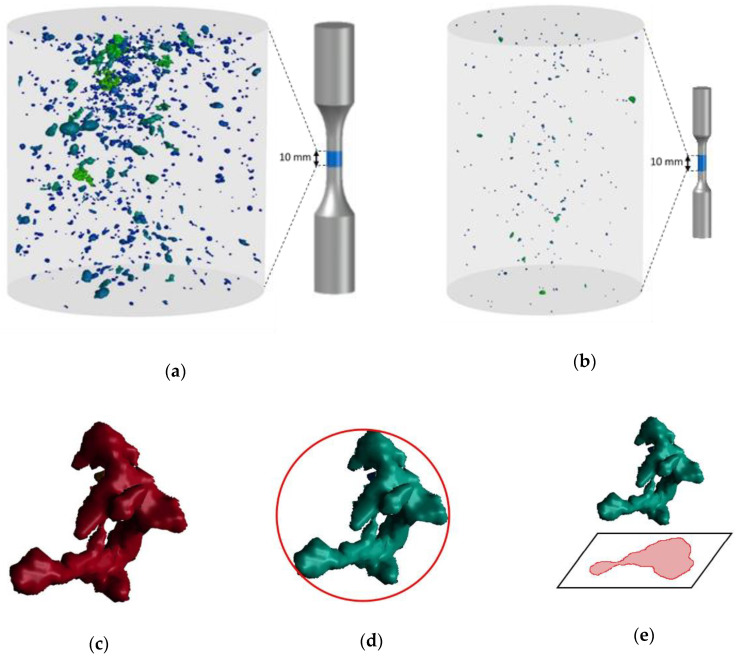
Examples of 3D images of defects in (**a**) large specimens, (**b**) small specimens, three different defect parameters characterizing the defects size, (**c**) volume of the defect ($\sqrt[{3}]{Volume}$), (**d**) max Feret diameter ($Feret_{max}$), and (**e**) area of the projected area on the plane perpendicular to the specimen axis ($\sqrt{A_{projected}}$).

In this study, two specimens at each porosity level in small and large specimens were chosen randomly for µCT scan and 3D micrographs of the defects within a small part of the specimen gage section (10 mm from the uniform gauge length) were reconstructed. In order to study the repeatability of defects in different specimens, three additional specimens in porosity levels one and three were examined in small and large specimens, respectively. In order to have high resolution 3D images, the voxel size (volumetric pixel) of (12 µm)^3^ was selected, which is far below the critical defect size in this material (between 50–100 µm), below which fatigue performance is not controlled by the defects [12,41]. Examples of 3D reconstructed images obtained for small and large specimens are illustrated in Figure 3a,b, respectively.

The 3D reconstructed images were analyzed using VG studio Max 3.3 (Volume Graphics GmbH). In analysis of defects, the maximum Feret diameter, volume, and the projected area of the defects on the plane perpendicular to the specimen’s axis were taken as defect parameters, characterizing the defects size. These defect parameters are shown in Figure 3c–e.

## 4. Defect Characterization

Defects can be formed in castings in variety of complex ways. Gas porosities are normally more or less spherical, while the shrinkage porosities have irregular and branched shape. Therefore, in order to quantify and compare the characteristics of defects, i.e., size and shape, it is required to define a defect parameter. 

In evaluating the defects using metallography, Murakami et al. [38] suggested that the square root of the projected area of defects on a plane perpendicular to the loading direction is a promising parameter to characterize the defects for evaluating the effect of both defect size and shape on fatigue strength. Furthermore, using finite element analysis, Gao et al. [24] showed that in a given projected area and local curvature, the maximum stress concentration factor of defects with different complexities are very close to each other. However, maximum Feret diameter, as the diameter of circumscribed circle (or sphere in 3D) on the defects is also widely used in literature for evaluating the effect of defects on fatigue behavior. In addition, the square root of the area of an ellipse fitted to the defects can also be a parameter to characterize the defects on polished surfaces. On the other hand, for evaluating the defects using X-ray µCT scan, three defect parameters can be used, including the projected area of the defects on the transverse plane, the maximum Feret diameter, and cubic root of volume of the defects. 

**Figure 4:**
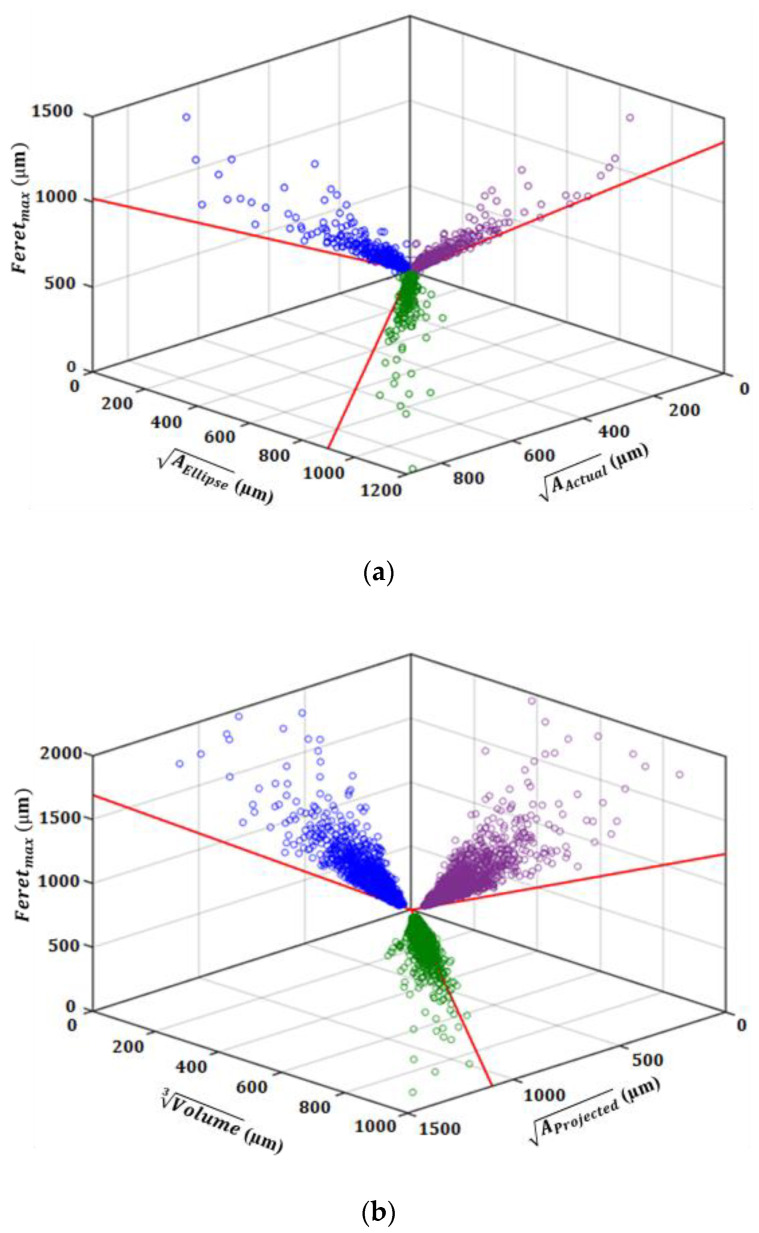
Comparison of different defect parameters; (**a**) metallography and (**b**) µCT scan evaluation. The solid diagonal lines pertain to the ideal spherical (circular) defect.

**Figure 5:**
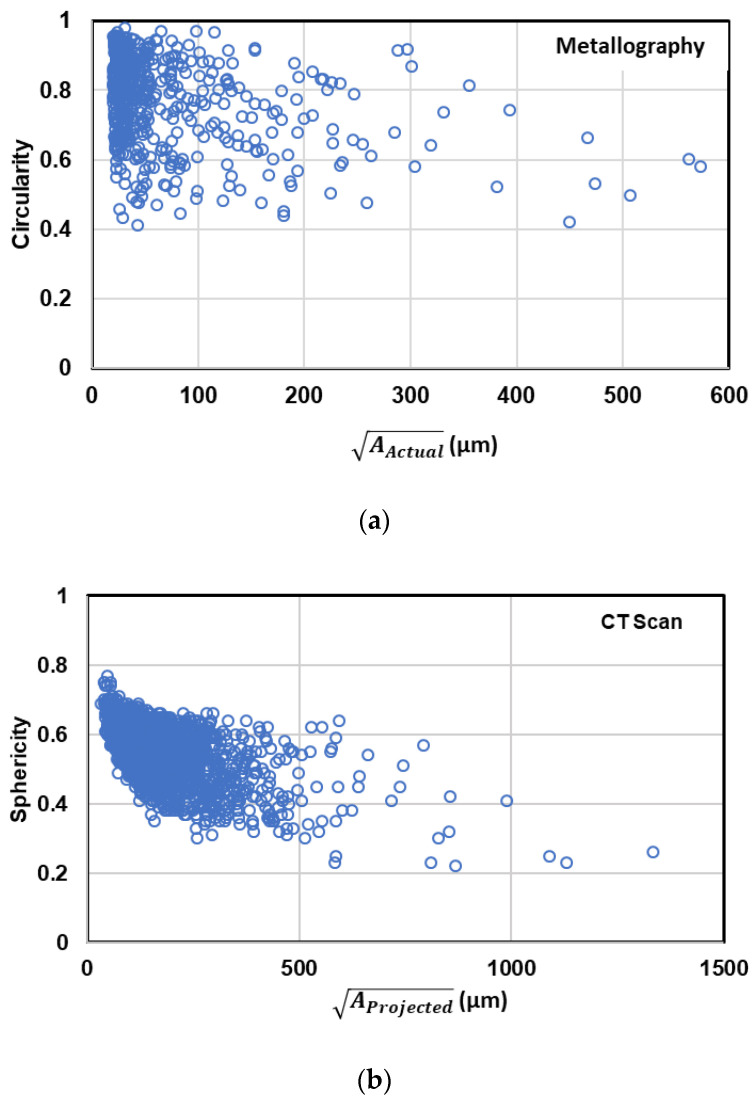
Comparison of the relation of defect size with sphericity or circularity of defect in (**a**) metallography and (**b**) µCT evaluations.

The comparison of different defect parameters and their deviation from an ideal circular or spherical defect for both 2D and 3D analysis is shown in Figure 4. It can be seen in this figure that the larger the pores, the more deviated they are from an ideal circular or spherical shape. This is due to the fact that larger pores are mostly shrinkage porosities with more complex and branched shape. The relation of size with sphericity (or circularity in 2D) of pores is shown in Figure 5. In this figure, sphericity is defined as the ratio of the surface area of a sphere with the same volume to the surface area of the actual defect. The same definition is also used for circularity using perimeter of the defects instead of surface area. 

Sphericity (or circularity in 2D) of defects is a measure of irregularity of defect shape which ranges between zero and one. As the sphericity decreases from one to zero, the irregularity of the defect increases from an ideal spherical defect to a highly irregular interconnected one. As can be seen in this figure, it is evident in both 2D and 3D evaluation that large defects are more tortuous with less sphericity (or circularity in 2D) than small ones. This fact is more evident in 3D results in comparison with the metallography data. This is because in 2D evaluation the tortuous defects may appear on metallography cross section as various complex shapes or a collection of small defects, due to sectioning of various dendritic arms during sample preparation. 

**Figure 6:**
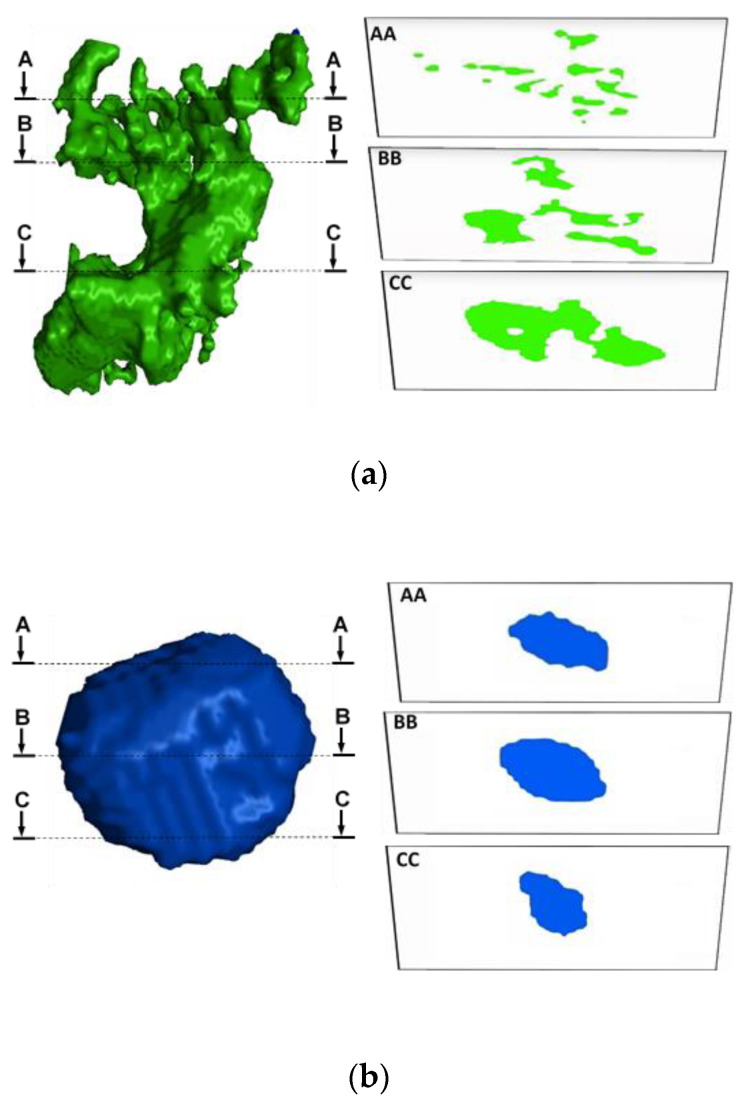
Illustration of (**a**) shrinkage and (**b**) gas porosity and their appearance on 2D surfaces.

Figure 6 shows how gas or shrinkage porosities may appear on random 2D surfaces, depending on the location of the cross section. As can be seen, the shape of shrinkage porosities on 2D surfaces depends on the location of the random cross section to a great extent. In the case of gas porosities which are more or less smooth, the dependency of defects shape on 2D surfaces is much less, in comparison to the shrinkage porosities. Furthermore, considering the comparison of different defect parameters in 2D scale (Figure 4a), it was observed that the average of $Feret_{max}/\sqrt{A_{Actual}}$ as well as $\sqrt{A_{Ellipse}/A_{Actual}}$ ratios for the defects larger than 100 µm are ~2 and ~1.3, respectively. This fact should be taken into account in evaluating and predicting the fatigue life of defect containing materials using different defect parameters. 

The effect of volume of the defects on fatigue performance of the materials depends on the orientation of defects with respect to the loading direction. Li et al. [31] showed that the projected geometrical features of defects on the plane perpendicular to the loading direction are more important than the actual length or volume of the defects. Therefore, in evaluating the defects using µCT data in the next sections, only the square root of the projected area of defects is taken into account as the defect parameter.

## 5. Defect Variability 

In this section the variability of defect distribution is evaluated statistically, including the variability of the defects between different specimens, as well as the variability of defects between porosity levels, along with the variability of defects in height and radial direction within the specimens. This analysis was performed using both metallography and µCT results while square root of the projected area was considered as the defect parameter. 

### 5.1. Variability of Pore Size Distribution in Specimens of the Same Size and Porosity Level

**Figure 7:**
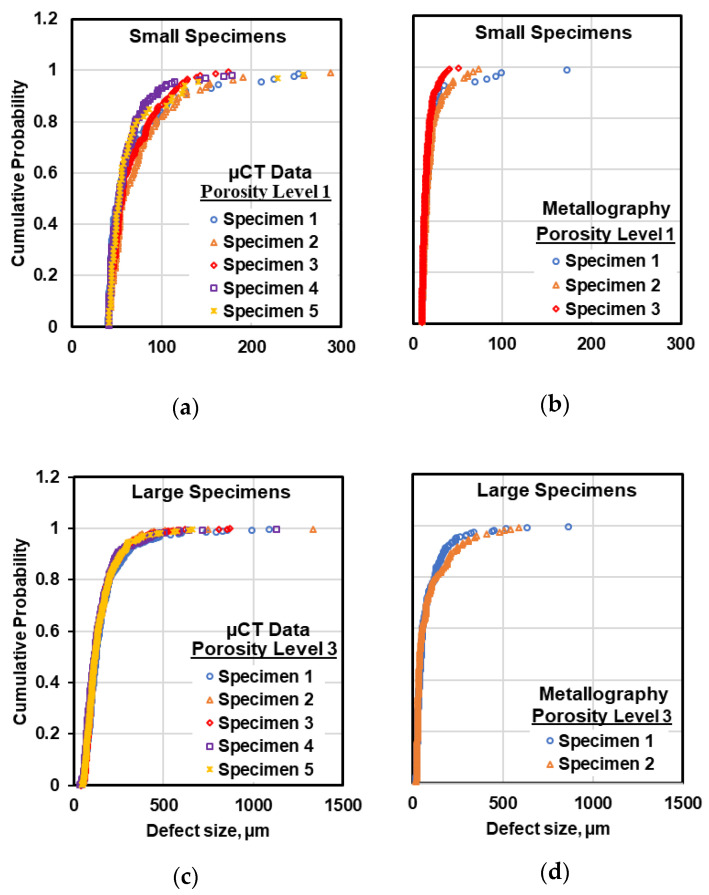
Examples of the defect distribution in different specimens of the size and porosity level using (**a**,**c**) µCT data and (**b**,**d**) metallography data.

For a given porosity level or specimens size, defect evaluation was conducted between two to five specimens. The comparison of defect size distributions between the specimens in a given size and porosity level was performed by comparing the cumulative probability distribution vs. defect size in different specimens. This comparison for small and large specimens at porosity levels one and three using the defect size data measured from both metallography and µCT micrographs are illustrated in Figure 7. As can be seen, the defect size distributions in different specimens are very similar to each other in both metallography and µCT evaluations. Similar comparisons were performed for other porosity levels as well and similar results were observed.

To compare the defect size distributions statistically, the Kolmogorov–Smirnov (K-S) test was applied, which is one of the most useful nonparametric methods for comparing two data sets. In the K-S test, the maximum value of the absolute difference between the two cumulative probability distributions (D) are calculated and compared to the critical value ($D_{\alpha }$), which is a function of sample size of the two data sets and level of significance (α). The null hypothesis that the two data sets come from the same underlying distribution is rejected if D is larger than the critical value, $D_{\alpha }$. Having applied this test, it was confirmed that in a given size and porosity level, the defect size distributions in different specimens pertain to one statistical population with a significance level of 0.05. This result was expected considering similar casting condition for all specimens. Therefore, the defect data measured on different specimens can be combined for further statistical analysis.

### 5.2. Variability of Defects Size Distribution between Pre-Defined Porosity Levels

**Figure 8:**
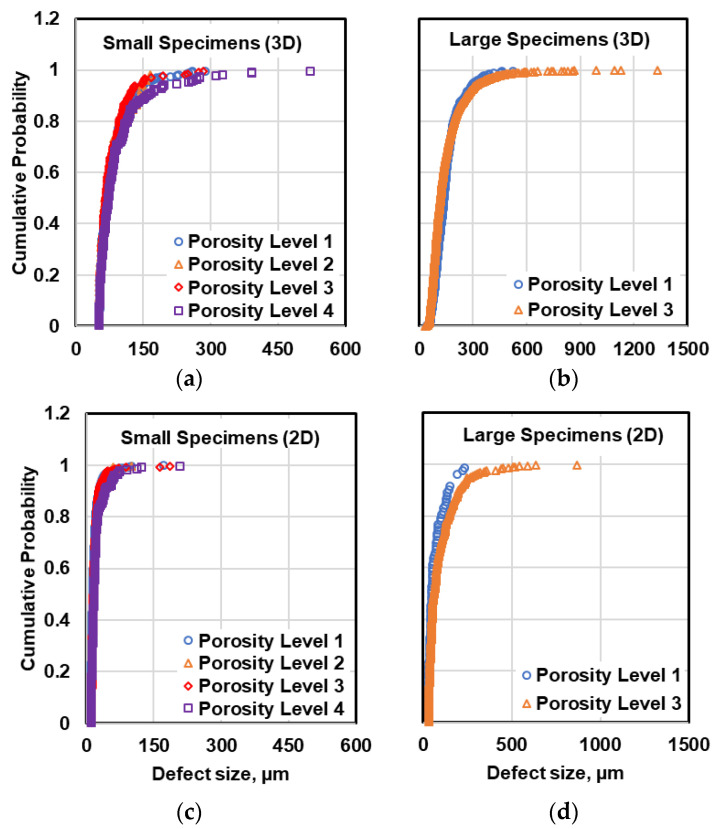
Comparison of defect distribution in different porosity levels measured by X-ray radiography in small and large specimens based on (**a**,**b**) µCT data and (**c**,**d**) metallography data.

**Figure 9:**
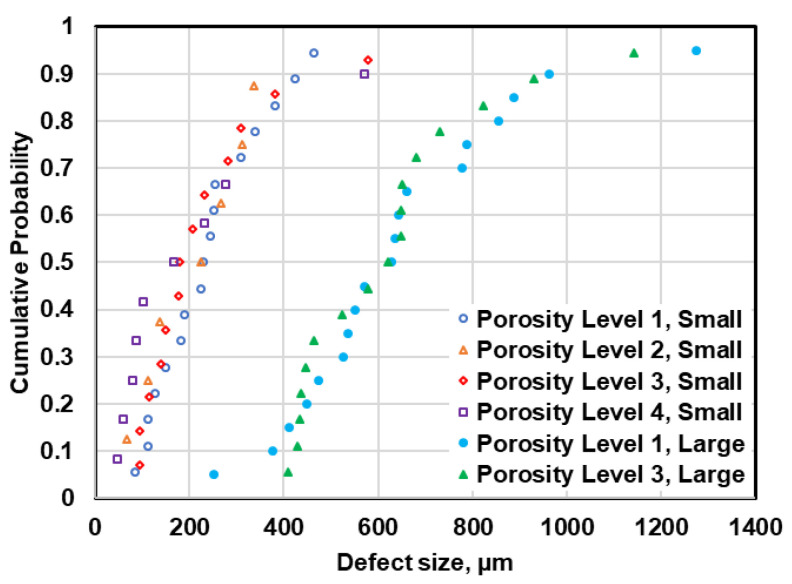
Comparison of defects distributions in different porosity levels based on observed defects on fracture surfaces.

The defect size distributions in different porosity levels can be compared for both small and large specimens, as shown in Figure 8. It can be seen that the defect size distribution plots obtained from µCT data for different porosity levels are very similar, while slight difference can be noticed between the distribution of defects in large specimens using 2D data. However, applying the K-S test on defect distributions in small and large specimens revealed that the defect data at four porosity levels belong to one statistical population. This fact can also be confirmed by comparing the distribution of fatal defects observed on fracture surfaces of the failed specimens at different porosity levels under uniaxial fatigue tests, illustrated in Figure 9. This figure reveals that the defect size distribution at different porosity levels are similar and no evident order can be seen in defect distributions. This suggests that using the common method of X-ray radiography often used in industrial application may not be reliable for defect evaluation. In X-ray radiography, the defects are evaluated based on their projections on two dimensional films, wherein the resolution is affected not only by the orientation of defects with respect to the source and the film, but also superposition of other defects. 

In Table 2, the range of maximum pore sizes within the specimens at different porosity levels measured from metallography and µCT micrographs are compared with the fatal pore sizes observed on fracture surfaces of failed specimens under uniaxial fatigue tests. It should be mentioned that the maximum pore size observed on metallography cross sections is associated to 24 cross sections of small specimens and 16 cross section of large specimens at a given porosity level. In the case of µCT scan, the maximum defects pertain to the measured ones within two to five specimens at each porosity level. The ranges of maximum defect size observed on fracture surfaces is associated with between 20 to 25 specimens at a given porosity level. As can be seen in Table 2, the maximum defects observed on fracture surfaces are larger than those measured from metallography sections or µCT micrographs. Further, no significant difference can be seen between the maximum fatal pore on fracture surface of the specimens at different porosity levels.

**Figure 10:**
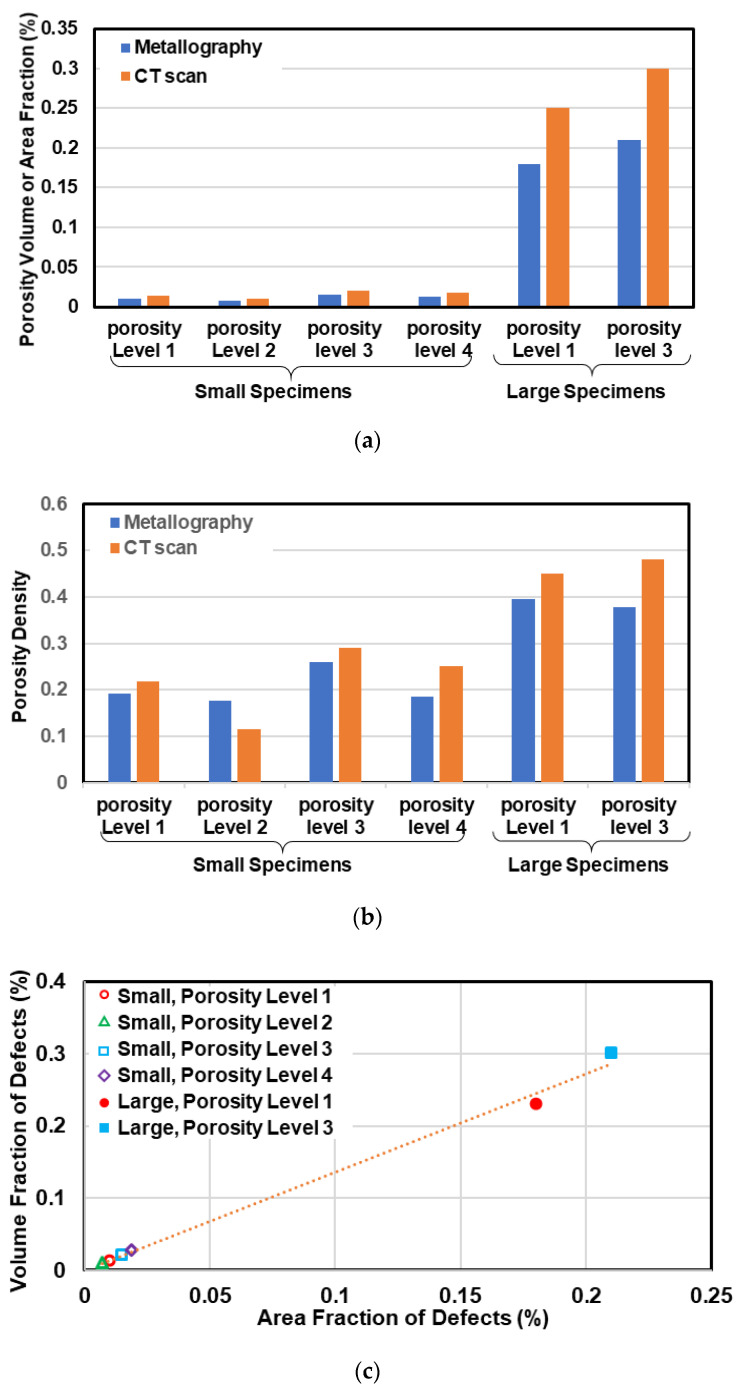
(**a**) volume or area fraction of defects, (**b**) porosity density per mm^3^ for CT scan and per mm^2^ for metallography data at each porosity level, and (**c**) volume fraction of defects from µCT scan vs. area fraction of defects by metallography.

The comparison between defect density as well as the volume (or area) fraction of defects at different porosity levels are shown in Figure 10a,b. Considering these figures, no significant difference can be seen, neither in defect density nor volume (or area) fraction of the defects within the specimens at different porosity levels. However, the volume (or area) fraction of defects as well as defect density of large specimens are significantly higher than those in small specimens. This suggests that as the casting section size increases, both the defect size and the population of defects are increased. 

The comparison of volume and area fraction of defects measured from µCT and metallography micrographs at different porosity levels is illustrated in Figure 10c. As can be perceived in this figure, the area fraction of defects measured from metallography sections are smaller than volume fraction of defects measured from µCT micrographs which is due to smaller size of defects on 2D micrographs in comparison to 3D defect size in µCT micrographs. This issue can also be seen in Table 2 by comparing the maximum defect range from metallography and µCT evaluations. 

### 5.3. Variability of Defects between Two Specimen Sizes

**Figure 11:**
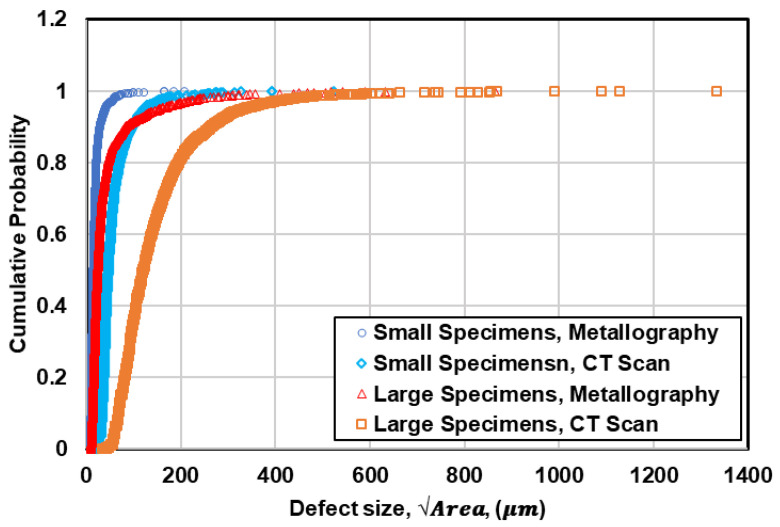
Comparison between defect distribution in small and large specimens using metallography and µCT data.

The comparison between defect distribution in small and large specimens using both metallography and µCT data is illustrated in Figure 11. As can be seen, the probability of having large defects in large specimens is significantly higher than that in small specimens based on both metallography and µCT data. This is in agreement with the observed fatal defects measured on fracture surfaces, shown in Figure 9. On the other hand, it can be clearly seen in Figure 11 that the defects observed on metallography cross sections are smaller than those measured from µCT micrographs, which is due to the dependency of defect analysis on location of metallographic cross sections in 2D evaluation of defects.

### 5.4. Variability of Defect Size in Specimen Height and Radial Directions 

**Figure 12:**
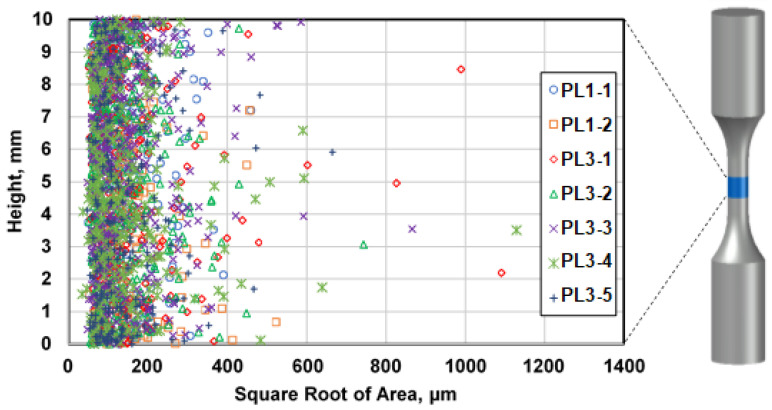
Distribution of defects along the height based on µCT data in large specimens. The symbols represent the defect data in different specimens at porosity levels 1 (PL1) and porosity level 3 (PL3).

In order to study the variability of defects along the height of the specimens, the distribution of defect size within the gauge length of the specimens using the defect data obtained through µCT is plotted vs. height in Figure 12. As can be clearly seen, the defects are distributed randomly throughout the gauge length and there is no evident order in distribution of defects along the height of the specimens throughout the gauge length. Similar results were obtained using defect data from metallography examinations.

**Figure 13:**
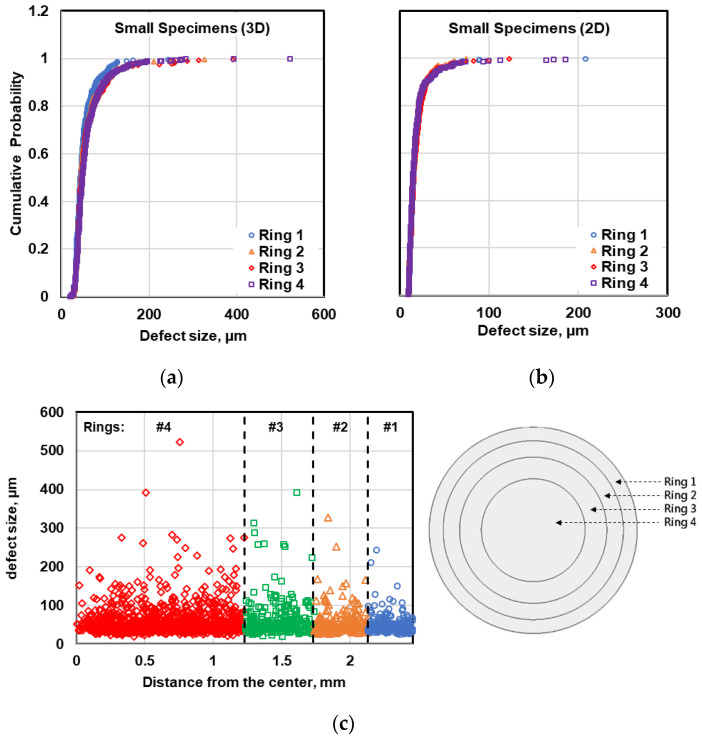
Comparison of the radial distribution of the defects within the cross section in small specimens using (**a**) µCT data, (**b**) metallography data, and (**c**) illustration of defect distribution in radial direction within small specimens.

**Figure 14:**
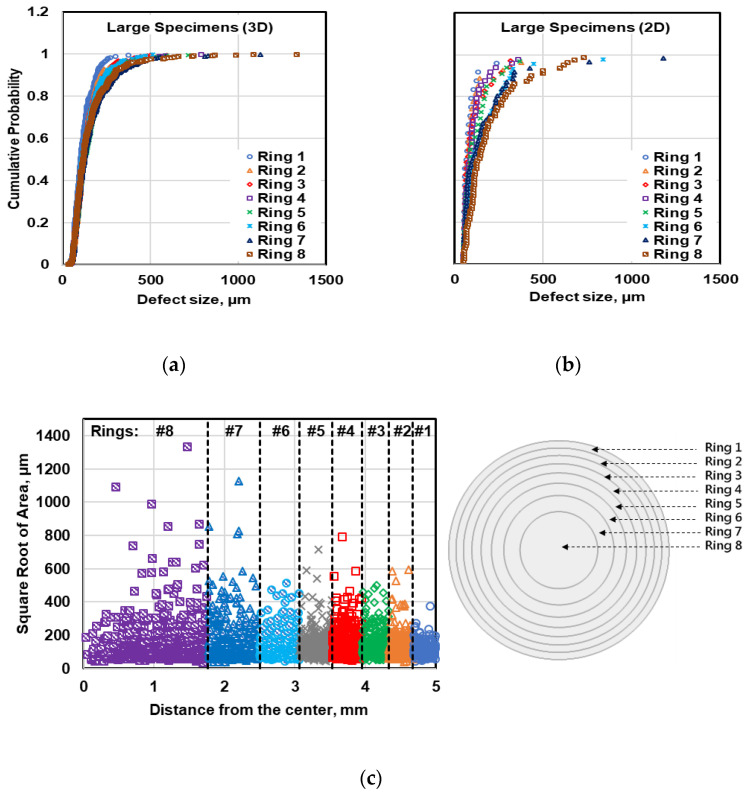
Comparison of the radial distribution of the defects within the cross section in large specimens using (**a**) µCT data, (**b**) metallography data, and (**c**) illustration of defect distribution in radial direction within large specimens.

To study the variability of defect distribution in the radial direction, each micrograph in both metallography and µCT evaluation was divided into four and eight subsections (coaxial rings) with equal volumes or areas in small and large specimens, respectively. Then, the radial distribution of the defects was studied by comparing the cumulative probability distribution of defects for different rings using 2D and 3D data as shown in Figure 13 and Figure 14 for small and large specimens, respectively. As can be seen in Figure 13, the central ring in small specimens are likely to have larger defects in comparison to the outer ring. However, the number of large pores at the inner rings is not significant to make evident difference in the cumulative probability distribution of defects at different rings. Applying the K-S test on these defect distributions also revealed that in small specimens the defect distribution at different rings are similar and the defects in all four rings can be considered as one statistical population. Therefore, it can be concluded that the defects in small specimens have been distributed randomly throughout the specimens. 

In the case of large specimens, however, there is an order in defect distributions at different rings, where the outer ring (Ring 1) contains the smallest defects and the most inner ring (Ring 8) has the largest defects. The dissimilarity of the defect distribution at different rings can be confirmed by applying the K-S test, using both 2D and 3D defect data (Table 3 and Table 4). Using the K-S test results based on metallography data, it can be concluded that defects within Rings 1–5 belong to one statistical population, while the defect within Rings 6–8 can be considered as another population. This classification is a little different when µCT data is used, where the outer region includes Rings 1–3 and the inner region contains Rings 4–8.

This difference between metallography and µCT data is associated with different appearance of the defects on metallography sections in comparison to the real defect size which can be measured from 3D micrographs. The difference between defect population in outer region of the large specimens from the inner part can be associated to different solidification rates in different regions of the specimens. The solidification rate in outer region of the specimens is higher which leads to less chance to have shrinkage porosity, while the cooling rate in inner region of the specimen is lower and the probability of having large shrinkage porosity is high. However, the lower thermal gradient from surface to center of small specimens results in a relatively uniform defect distribution in the entire cross section. Having combined the defect size data in similar rings in large specimens, the defect distribution of outer region and inner region can be obtained. Therefore, the defect data in Rings 1–3 and Rings 4–8 have been combined. 

**Figure 15:**
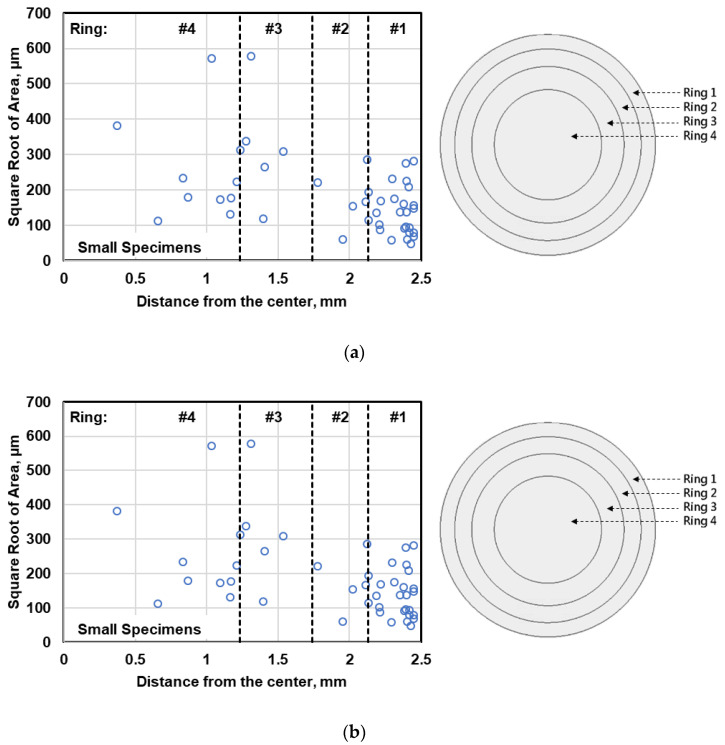
Comparison of the radial distribution of the fatal defects observed on fracture surfaces in terms of their distance from the center in (**a**) small and (**b**) large specimens.

The distributions of the fatigue crack initiating defects measured on the fracture surface of the small and large specimens are demonstrated in Figure 15. Considering this figure, it can be inferred that the fatigue crack may originate from both outer and inner regions of the specimens. It is believed that the large defects at or near the specimen surface are more detrimental in fatigue. For defects of the same size, the stress concentration around a defect at the surface is higher than that of an internal one, while at a given distance from the surface, the stress concentration around large defects are higher than that of small defects [24]. Therefore, there is competition between the defects in terms of size and location.

## 6. Application of Extreme Value Statistics to Estimate the Maximum Defects 

As the fatigue cracks are most likely to originate from larger defects relative to smaller ones, the fatigue properties of defect containing materials will be governed by the defects on the upper tail of the defect probability distribution. Therefore, in order to evaluate and predict the fatigue life of the specimens it will be advantageous to know the maximum defect size which may exist within the specimen or component. The maximum defect size is unlikely to be observed by metallography examination through random cross sections. On the other hand, using µCT scan on a specimen is costly but will provide accurate defect size in the scanned region of the specimen. However, even in a given cumulative probability distribution the maximum defect may differ from one specimen to another. Therefore, in order to estimate the maximum defect size within the desired volume, extreme value statistics can be used which has shown to be very useful in predicting the maximum defect size based on metallography and µCT data. The estimated maximum defect can, then, be an input to fatigue life prediction models to estimate the fatigue life under the desired loading condition in fatigue design.

Regarding applying extreme value statistics on metallography data, block maxima is the most common approach in which several subsections with the same area, *S_0_*, are inspected, and only the maximum defect within each inspected area is recorded. On the other hand, for estimating the maximum defect size using µCT data peak-over-threshold approach is often used which consists of considering all the defects within the scanned volume above a given threshold, *u*, therefore no important information is lost. In this study, the BM and POT approaches are followed to estimate the maximum defect within the specimens using metallography and µCT data, respectively. The accuracy of each method is, then, verified by comparing the estimated maximum defect with the maximum fatal defects observed on fracture surfaces.

### 6.1. Block Maxima (BM) Approach

To use BM approach to estimate the maximum defect size within the specimens several standard control areas are inspected on metallography micrographs and the maximum defects in each control area is determined. The controlled areas used for small and large specimens were 2.37 and 4.91 mm^2^, respectively. In each control area, the maximum defect was determined, and the maxima data points were plotted as cumulative probability distribution. Extreme value distribution functions were, then, fitted into the maxima data point. It should be mentioned that in predicting the maximum defect size within the specimens in addition to extreme value (Gumbel) distribution function, EV, which is very common to be used in estimating maximum defect size, GEV distribution function was also used. Therefore, both EV or GEV distribution functions are fitted to the defect size cumulative probability distributions using maximum likelihood (ML) method and the corresponding parameters including location, scale, and shape parameters were determined (see Equations (1) and (3)). 

The maximum defect size within the specimens is, then, the size of a defect associated with the return period, *T*, defined by Equation (2). Therefore, the maximum defect size within the specimens, associated to probability of P=1−1/T, was estimated using Equations (7) and (8) based on EV and GEV distributions, respectively:

(7) $$x_{EV}=\mu +\sigma \left[-\ln\left(-\ln\left(1-\frac{1}{T}\right)\right)\right],$$

(8) $$x_{GEV}=\mu -\frac{\sigma }{\xi }\left[1-{\left(-\ln\left(1-\frac{1}{T}\right)\right)}^{-\xi }\right].$$

**Figure 16:**
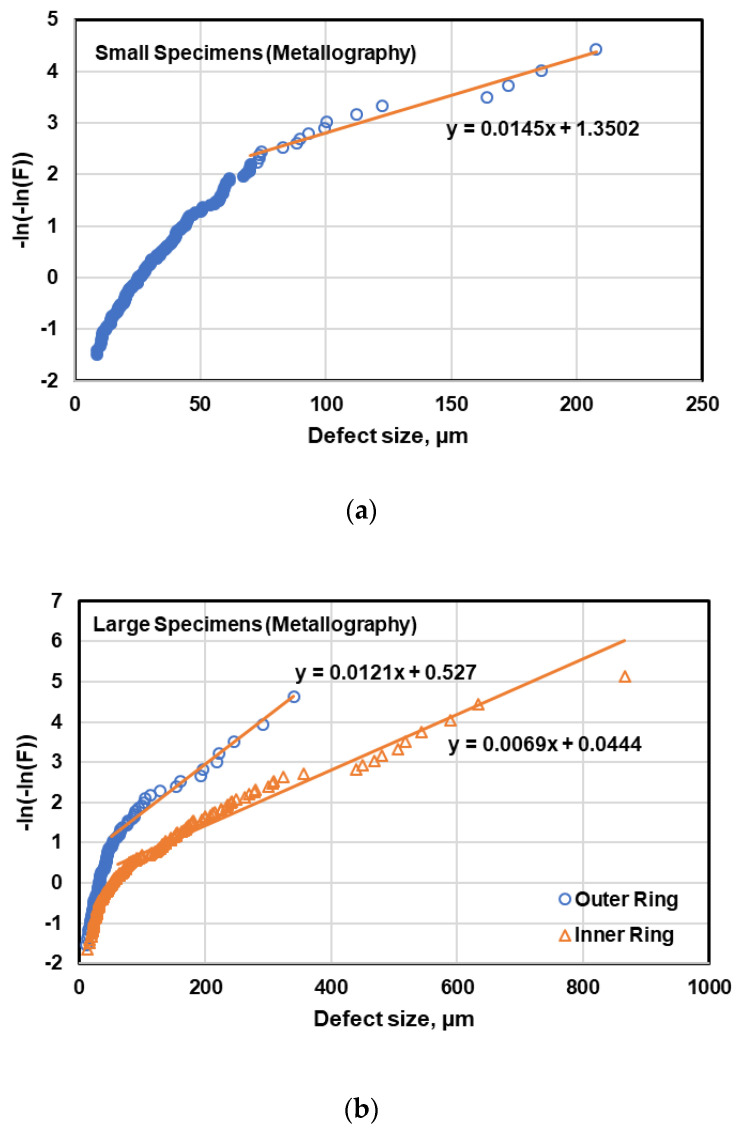
Extreme value (EV) plot of defect data for (**a**) small and (**b**) large specimens.

For estimating the maximum defect size using EV function, the data points are usually plotted as −ln(−ln(P)) vs. defect size, which is expected to be linear according to Equation (3). However, it was observed that when the defect data are plotted based on EV distribution function all the data points are not aligned on a straight line (see Figure 16). So, it can be inferred that EV distribution is not representative of the entire pore distribution. This nonlinearity of the data points can be attributed to presence of two kinds of porosity, i.e., small and large [3]. Therefore, a threshold for defect size was defined from EV distribution plots where the inflection occurs, which is in the range of 40–80 µm. Then, the defects with sizes above this threshold were considered in the evaluation. This approach was also followed in [6,23]. However, there are two challenges in this approach. Firstly, elimination of the defect with the sizes below the threshold may leave only few data points and prediction of the maximum defect size based on extrapolating the line fitted to few data points may not be reliable [6,39]. In addition, assuming a linear relation and extrapolating may cause a significant error in estimation of the maximum defect size. 

**Figure 17:**
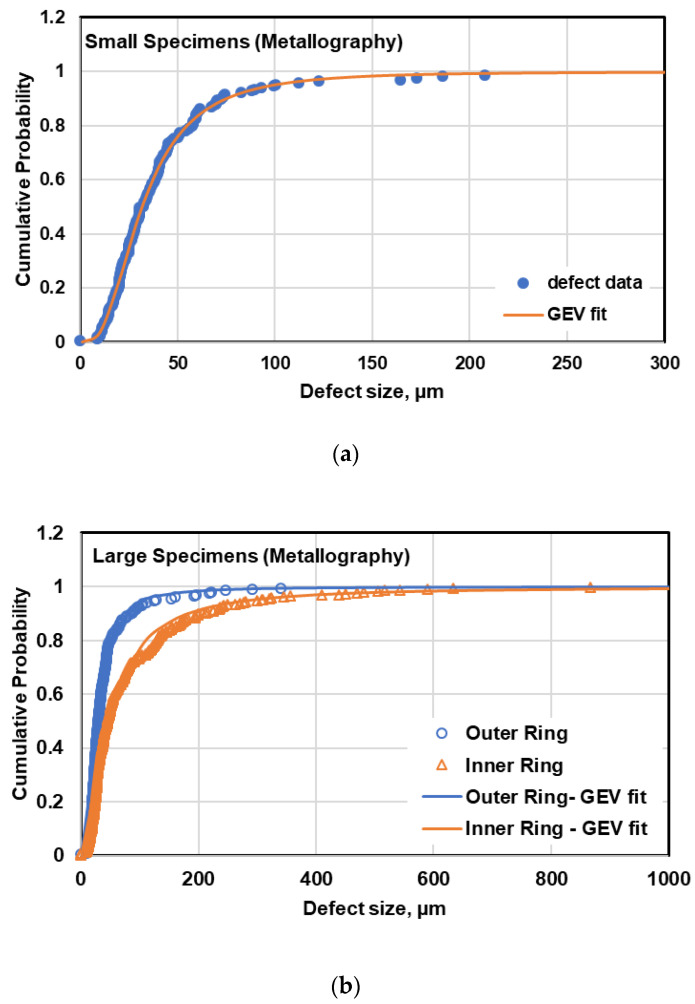
Generalized extreme value (GEV) plot of defect data for (**a**) small and (**b**) large specimens.

The maximum defect size can also be estimated using the generalized extreme value by fitting the GEV function to the cumulative probability distribution of the defects, shown in Figure 17. In this way, the GEV parameters can be determined using maximum likelihood method and the maximum defect size is computed by Equation (8). The advantage of this method is that all data points can be considered in prediction, as opposed to the EV distribution function in which the line was fitted only to the defects larger than a threshold.

The maximum defect size predicted by EV and GEV functions is compared with the maximum size of fatal defects observed on fracture surfaces of 80 small specimens and 40 large specimens in Table 5. As can be seen in this table, the prediction of maximum defect within the specimens using GEV distribution function is in very good agreement with the maximum observed defects on fracture surfaces. The estimate of EV distribution function is lower than that of GEV function, which is due to the assumption of linear relation of the data and extrapolation. This can also be attributed to a limited number of data points larger than the threshold, especially in the case of outer ring of large specimens, as shown in Figure 16b. 

It should be noted that in small specimens in which the defects are distributed randomly, the maximum defect in the entire cross section was computed. However, in large specimens where the defect distributions in outer and inner rings are different the maximum defects in each ring was calculated. For computing the maximum defect in the entire cross section of large specimens the concept of weakest link theory can be applied. 

According to the weakest link theory, in a system consisting of i elements each with probability of survival $P_{i}$, the probability of survival of the whole system will be the product of survival probability of all the elements:

(9) $$P=\overset{n}{\underset{1}{\prod }}P_{i}=P_{1}.P_{2}\ldots P_{n}.$$

In specimens or components containing defects, the probability of having defects smaller than a critical size corresponds to the cumulative probability distribution of defects:

(10) $$P_{X}\left(x\right)=P\left(X\leq x\right),$$

where the critical defect size, x, can be defined as the size of a defect that the materials will not fail at a given cyclic stress level. Therefore, the cumulative probability distribution of defects can be considered as survival probability. As the defect distributions in different regions of large specimen are different and do not belong to the same statistical population, the probability distribution of the whole specimen can be obtained using the weakest link theory (Equation (9)). 

In nearly all the published works on evaluating defects in cast Al alloys it has been assumed that the defects are distributed randomly throughout the cross section. This assumption can be true when the size of the specimens is small, while in large specimens or components in which the solidification rates in different regions are different defects and distributed nonuniformly. Therefore, the assumption of having uniform distribution may not be true and predicting the maximum defect based on this assumption may result in erroneous results. As a result, in order to study how the assumption of uniform defect distribution affects the estimate of the maximum defect size, the extreme value statistics was also applied to the whole cross section as one statistical population.

The estimated maximum defect when the whole cross section is considered as one statistical distribution and the one obtained by utilizing the weakest link theory for large specimens are reported in Table 5. As can be seen in this table, the assumption of uniform defect distribution results in overestimated prediction for maximum defect size using GEV approach, while prediction based on weakest link theory is in very good agreement with the maximum defects observed on fracture surfaces.

### 6.2. Peak-Over-Threshold Approach

For estimating the maximum defect size using µCT data, POT approach is often used. In this approach all the defects within the scanned volume above a given threshold, *u*, are considered. The GPD function is then fitted to the cumulative probability distribution of defects using ML method to determine the shape, γ, and scale, σ, parameters (Equation (5)). 

**Figure 18:**
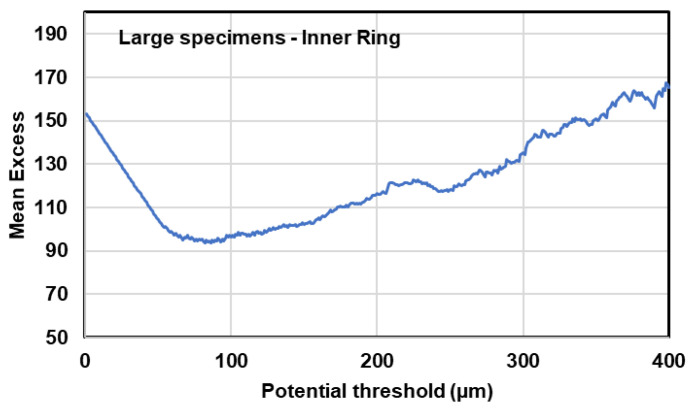
Mean excess plot using µCT data in large specimens in inner ring.

The threshold value for defect size can be determined using mean excess plot, selecting the point over which the mean excess of the large data becomes linear. Using the µCT data, mean excess plots for small and large specimens was plotted, through which the threshold defect size of 40 µm and 100 µm was chosen for small and large specimens, respectively. As an example, the mean excess plot for large specimens in inner ring is demonstrated in Figure 18.

Having determined the threshold defect size, the shape and scale parameter in GPD distribution function for small and large specimens, the maximum defect size within the specimens was computed using:

(11) $$x_{GPD}=u-\frac{\sigma }{\gamma }\left(1-T^{\gamma }\right),$$

where the return period, *T*, was calculated using Equation (6). The estimated maximum defects within small and large specimens are summarized and compared with the estimates of BM approach in Table 5. As can be seen, the prediction of maximum defects based on µCT data are in very good agreement with the maximum defects observed on fracture surfaces. Comparing the predictions of maximum defect based on metallography and µCT scan reveals that the predictions based on metallography data are underestimated relative to those of µCT data, while the discrepancy between the predictions is not significant. Lower estimation of maximum defect size based on metallography data is associated with the dependency of metallography on the location of the cross sections. This effect can be reduced by increasing the number of metallography sections, as was performed in this study. 

## 7. Summary and Conclusions

As the mechanical properties of cast components can be significantly affected by existing defects, evaluation of defects within specimens or components is of great importance in design. In this work, A356 aluminum specimens of two sizes were produced by HPDC, as an illustrative material containing defects. Initial evaluation of defects was carried out using X-ray radiography, through which the specimens were classified into four porosity levels with regards to the maximum detected defects size. The defects were then characterized using metallography as well as micro-computed tomography techniques. The variability of defects between the specimens of two sizes as well as different porosity levels were studied statistically. The distributions of defects based on location within the specimens were also analyzed. Moreover, the maximum defect size within the specimens was estimated using extreme value statistics based on both 2D and 3D defects data. The following conclusions are drawn from these analyses:

1. Cumulative probability distributions of defects based on 2D and 3D evaluations as well as the measured defects size from the fracture surfaces of the specimens at different pre-defined porosity levels suggest that the common method of X-ray radiography often used in industrial application may not be reliable for defect evaluation.
2. Examining different defect characteristics in metallography examinations as well as µCT scan evaluations revealed that as the size of pores increases, they deviate more from ideal spherical (or circular in 2D) defect, indicating reduced sphericity (or circularity in 2D) of pores with increasing defect size.
3. Studying the variability of defects in radial distribution revealed that the pore distribution in the specimens produced by high pressure die cast may not be random throughout the cross section, especially in large section sizes. In addition, as the casting section size increases, both the defects size and density also increase.
4. Evaluating defects using µCT scan is very accurate but not always accessible, contrary to metallography approach which is accessible and cost effective while defects evaluation through metallography depends on the location of random cross section and the pores measured on metallography micrographs are smaller than those measured through µCT scan. However, increasing the number of metallography cross sections improves the accuracy of 2D measurements.
5. Applying extreme value statistics to metallography data using GEV distribution function could estimate the maximum defects in reasonable agreement with the maximum observed defects on fracture surfaces. The estimate of maximum defect using POT approach to the µCT data was also in very good agreement with the maximum observed defect.
6. Although EV distribution function is commonly used for estimating the maximum defect size using metallography data, the estimated maximum defect using this approach was underestimated in comparison to the maximum observed defect on fracture surfaces.
7. To estimate the maximum defect size of the entire large specimen where the defects are not distributed randomly the weakest link theory was applied and the prediction of the weakest link theory was in better agreement with the maximum defect size on fracture surfaces, in comparison with the prediction using the assumption of random distribution throughout the cross section.

## Figures and Tables

**Table 1:** Classification of specimens based on the maximum defects detected in X-ray radiography as well as the volume fraction of the defects in small and large specimens measured from X-ray micro-computed tomography (µCT) scan results.

Porosity Level	Max Defect Size (µm)	Volume Fraction of Defects (%)	
		Small Specimens	Large Specimens
1	0–62	0.013	0.251
2	63–124	0.010	0.193
3	125–184	0.021	0.317
4	185–250	0.018	0.215

**Table 2:** Comparison of observed maximum defect size in different porosity levels (the measurements are in µm).

Method	Small Specimens				Large Specimens	
	Porosity Level 1	Porosity Level 2	Porosity Level 3	Porosity Level 4	Porosity Level 1	Porosity Level 3
Metallography	51–173	59–104	88–207	112–208	153–234	589–866
µCT	175–288	155–167	243–283	391–522	461–520	662–1333
Fracture surface	84–463	67–337	93–578	47–571	252–1274	408–1141

**Table 3:** Kolmogorov–Smirnov (K-S) test results for comparing defect distribution in large specimens at different “Rings” using metallography data.

Location	Ring 1	Ring 2	Ring 3	Ring 4	Ring 5	Ring 6	Ring 7	Ring 8
Ring 1	-	**√**	**√**	**√**	**√**	**×**	**×**	**×**
Ring 2	**√**	-	**√**	**√**	**√**	**√**	**×**	**×**
Ring 3	**√**	**√**	-	**√**	**√**	**√**	**√**	**×**
Ring 4	**√**	**√**	**√**	-	**√**	**√**	**√**	**×**
Ring 5	**√**	**√**	**√**	**√**	-	**√**	**√**	**√**
Ring 6	**×**	**√**	**√**	**√**	**√**	-	**√**	**√**
Ring 7	**×**	**×**	**√**	**√**	**√**	**√**	-	**√**
Ring 8	**×**	**×**	**×**	**×**	**√**	**√**	**√**	-

**Table 4:** K-S test results for comparing defect distribution in large specimens at different “Rings” using CT scan data.

Location	Ring 1	Ring 2	Ring 3	Ring 4	Ring 5	Ring 6	Ring 7	Ring 8
Ring 1	-	**√**	**√**	**×**	**×**	**×**	**×**	**×**
Ring 2	**√**	-	**√**	**√**	**√**	**√**	**×**	**×**
Ring 3	**√**	**√**	-	**√**	**√**	**√**	**√**	**√**
Ring 4	**×**	**√**	**√**	-	**√**	**√**	**√**	**√**
Ring 5	**×**	**√**	**√**	**√**	-	**√**	**√**	**√**
Ring 6	**×**	**√**	**√**	**√**	**√**	-	**√**	**√**
Ring 7	**×**	**×**	**√**	**√**	**√**	**√**	-	**√**
Ring 8	**×**	**×**	**√**		**√**	**√**	**√**	-

**Table 5:** Comparison of the maximum defect estimated by block maxima (BM) approach using metallography (2D) data as well as those estimated by peak-over-threshold (POT) approach using µCT (3D) data with the maximum fata defects observed on fracture surfaces (the unit of the estimates is in µm).

Estimating Approach	Small Specimens	Large Specimens			
		Outer Ring	Inner Ring	Whole Surface	Weakest Link
GEV (2D)	517	941	1232	1571	1362
EV (2D)	358	561	1022	1061	1113
GPD (3D)	602	1035	1303	1383	1426
Observed	578	963	1274	1274	1274
